# Evaluating the efficiency of mandibular molar protraction using Herbst appliances versus temporary anchorage devices: a retrospective case-controlled study

**DOI:** 10.1186/s40510-024-00533-3

**Published:** 2024-09-02

**Authors:** Ishita Z. Taneja, Guihua Zhai, Neal D. Kravitz, Bill Dischinger, Mark Johnston, Chung-How Kau, Ejvis Lamani

**Affiliations:** 1 Department of Orthodontics, School of Dentistry, University of Alabama at Birmingham, 1919 7th Avenue South, SDB 313, Birmingham, AL 35294-0007 USA; 2 Center for Clinical and Translational Science, University of Alabama at Birmingham, Birmingham, AL USA; 3 Private Practice, South Riding, VA USA; 4 Private Practice, Lake Oswego, OR USA; 5 Private Practice, Marietta, GA USA

**Keywords:** Tooth agenesis, Anchorage, Molar protraction, Herbst, TAD

## Abstract

**Background:**

Mandibular second premolar agenesis is a common problem in orthodontics and is often treated in conjunction with maxillary counterbalancing extractions. However, in cases without maxillary crowding or dental protrusion, space closure may pose challenges leading to compromised occlusal results or patient profile. Multiple techniques have been described to treat these patients; nevertheless, there is a paucity of data comparing effectiveness of space closure utilizing various anchorage techniques. The goal of this study is to assess the effectiveness of the Herbst device during mandibular molar protraction and compare it to the use of temporary anchorage device (TADs) in patients with mandibular second premolar agenesis.

**Materials and methods:**

This retrospective study included 33 patients with mandibular premolar agenesis treated without maxillary extractions. Of these patients, 21 were treated with protraction Herbst devices and 12 with TADs. Changes in molar and incisor positions, skeletal base positions and occlusal plane angulations were assessed on pretreatment (T0) and post-treatment (T1) lateral cephalograms. Scans/photographs at T0 and T1 were used to evaluate canine relationship changes representing anchorage control. Space closure and breakage/failure rates were also compared. Data was analyzed with paired and unpaired t-tests at the significance level of 0.05.

**Results:**

Within the Herbst group, changes in mandibular central incisor uprighting and mandibular molar crown angulations were statistically significant. However, no significant differences were noted between the Herbst and TAD groups. Protraction rates as well as overall treatment times were comparable (0.77 mm/month vs. 0.55 mm/month and 3.02 years vs. 2.67 years, respectively). Canine relationships were maintained or improved toward a class I in 82.85% of the Herbst sample, compared to in 66.7% of the TAD sample. Emergency visits occurred in 80.1% of the Herbst group, with cementation failures or appliance breakages as the most common reasons.

**Conclusion:**

The Herbst device could be a viable modality in cases with missing mandibular premolars where maximum anterior anchorage is desired, or if patients/parents are resistant to TADs. Furthermore, they could be beneficial in skeletal class II patients with mandibular deficiency who also need molar protraction. However, the increased incidence of emergency visits must be considered when treatment is planned.

## Background

Tooth agenesis (TA), or the congenital absence of one or more teeth, is a frequently encountered dental anomaly. Mandibular second premolar agenesis has a prevalence of 2.91 to 3.22% [1], and is unilaterally absent in 60% of cases [2–4]. When treating patients with congenitally missing mandibular second premolars, the orthodontist must make a timely decision regarding the deciduous tooth: be it extraction and space closure, or maintenance with the possibility of tooth loss in the future. The presence of ankylosis, root health of the deciduous tooth and remaining jaw growth are some of the factors that influence this decision [5]. Electing to extract the primary and close space is advantageous when there is crowding within the arch or a protrusive profile, since the space obtained can be used for this purpose [5]. However, in a situation where the profile is normal and there is no crowding, extra or intra-oral anchorage must be supplemented during space closure. This anchorage will prevent unwanted effects such as excessive uprighting/retraction of incisors and its negative impact on the soft tissue [5, 6].

Temporary anchorage devices (TADs) are often used in orthodontic treatment to support anchorage requirements. Sandler et al., compared anchorage reinforcement provided by palatal TADs with headgears, and found no statistically significant differences in overall PAR scores, treatment times or patient acceptance [7]. Furthermore, the use of TADs to protract posterior teeth has been documented in literature, primarily in the form of case reports. While most publications involved the use of skeletal anchorage to protract teeth into a first molar space [8–11], the use of mini implants to protract teeth in cases with missing premolars has also been documented [12, 13]. However, directly protracting a mandibular molar from a TAD placed laterally and inferiorly to the archwire may result in unwanted side effects such as a lateral crossbite and open bite tendency [14].

While traditionally utilized to correct a sagittal skeletal discrepancy in orthodontic patients [15], the use of fixed functional appliances as means to increase mandibular anterior anchorage and aid in posterior tooth protraction has also been documented [16]. Fiorentino and Melsen described using a Herbst appliance in conjunction with fixed bonded appliances to protract a mandibular molar in a patient with a congenitally missing premolar [16]. Zimmer and Rottwinkel, on the other hand, evaluated space closure using the Jasper Jumper as anchorage reinforcement in patients with bilateral aplasia of the mandibular premolars [17]. The Forsus device has also been used to reinforce anchorage during protraction of mandibular teeth [18, 19]. While the use of the Herbst as a Class II corrector has been extensively documented, its application in molar protraction is limited and further studies are needed [13].

The purpose of this study was to evaluate the Herbst protraction device in mesialization of mandibular molars in patients with congenitally missing mandibular second premolars. This study also aimed to compare the data from the protraction Herbst device with data obtained from TAD utilization for mandibular molar protraction. The hypothesis was that there would be no difference in the efficiency of space closure when using the Herbst device or TADs.

## Materials and methods

### Study design and patient sample

This retrospective case-controlled study was approved for exemption from the University of Alabama at Birmingham Institutional Review Board (IRB-300,008,326). Patients included in this study presented with unilateral or bilateral congenitally missing mandibular second premolars and were treated with fixed appliances and either the protraction Herbst device or TADs as an adjunct. Patients treated with maxillary counterbalancing extractions, those with other missing teeth excluding third molars, craniofacial discrepancies or syndromes, patients treated with orthognathic surgery, and charts with incomplete records were excluded. A total of 33 patients were included in the study, 21 patients in the Herbst group, and 12 in the TAD group. Orthodontic records of patients treated to completion with mandibular molar protraction using the Herbst device for anchorage reinforcement were provided from the orthodontic clinics of two providers, M.J. and N.K, who use 022 twin bracket systems. The data for the cases where TADs were used for protraction were obtained from another provider, B.D, who uses self-ligating 022 bracket system.

### Orthodontic appliances used for space closure

**Fig. 1:**
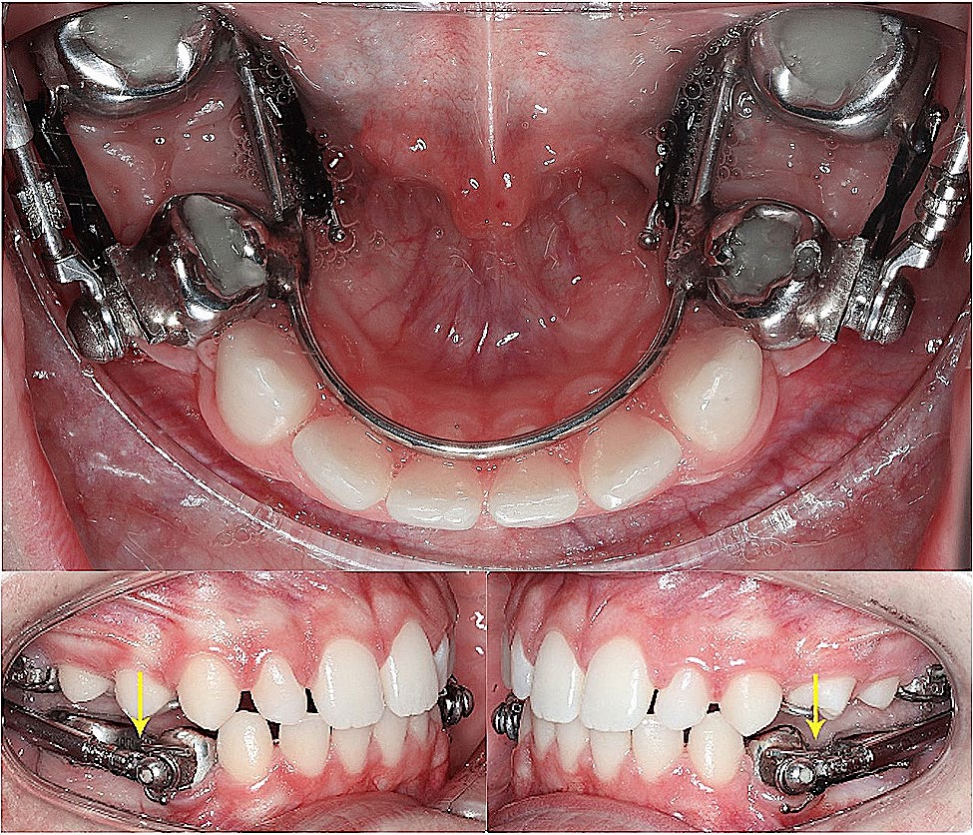
Occlusal and buccal views of the protraction Herbst device. Arrows showing the pivots for the Herbst rods present on the mandibular first premolar crowns [20]

The protraction Herbst device is a modification of the Herbst, where the arms connect from bands on the maxillary first molar to bands or crowns on the mandibular first premolar. The mandibular first molars slide along extensions of an 040 or 045 lingual arch soldered on the premolar bands. Protraction occurs by forces applied via elastomeric chains attached to hooks on the buccal and lingual of bands on the mandibular first premolar and molar (Fig. 1). Patients were recalled every 2–3 months. Mandibular second molars were not bracketed during the protraction phase, and allowed to drift mesially with the first molars. After removal of the Herbst, brackets were placed on all posterior teeth and laced together to maintain space closure. No wire cinching was used during protraction.

**Fig. 2:**
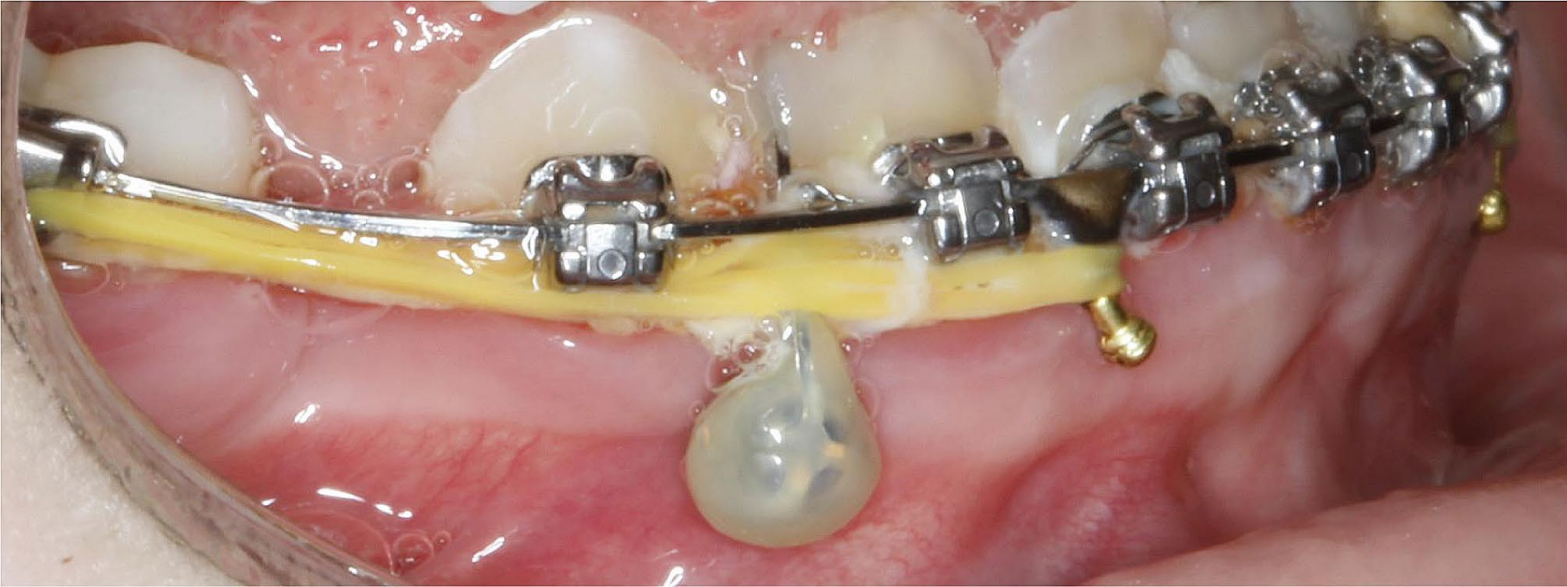
Mandibular TAD placement in attached gingiva occlusal to the muco-ginigival margin between lower right canine and premolar. SS wire was bonded to the TAD head and the adjacent premolar, and secured with composite resin for indirect rigid anchorage to the TAD

For protraction via TADs, the protocol used in this study involved buccal placement of an 8 mm TAD, between the mandibular canine and first premolar on the side affected by agenesis. The TAD was placed in attached gingiva occlusal to the muco-gingival margin. A piece of 16 × 25 SS wire was bonded to the TAD head and the adjacent premolar, and secured with composite resin for indirect rigid anchorage to the TAD (Fig. 2). Sliding mechanics were carried out with elastomeric chains to protract the molar while in uncinched 16 × 25 SS wires.

### Variables and data collection

**Fig. 3:**
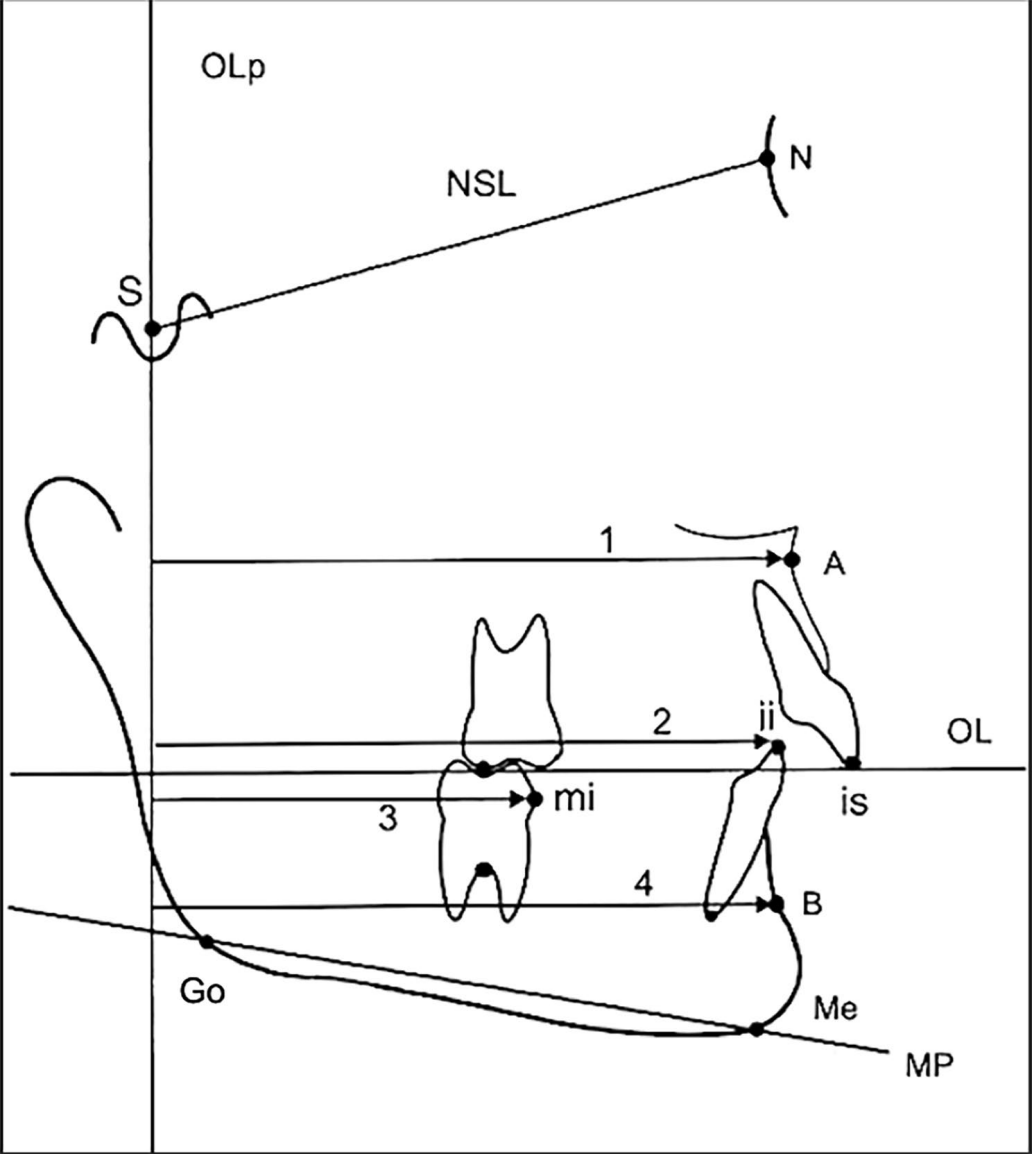
A representation of the lateral cephalogram tracing used for linear sagittal measurements. 1- A-OLp: shortest distance between A point and OLp, 2- ii-OLp: shortest distance between incisal tip of most prominent mandibular central incisor and OLp, 3- Mi-OLp: shortest distance between most mesial point on the crown surface of the mandibular first molar and OLp, 4- B-OLp: shortest distance between B point and OLp

Age and gender were collected for each patient in the study. From the pre- and post-treatment records of these patients we used lateral radiographic cephalograms to identify 12 hard tissue anatomical landmarks, as seen in Fig. 3. Three horizontal reference lines were then constructed using some of these landmarks: the Nasion-Sella line (NSL), the Occlusal Line (OL) and the Mandibular Plane (MP). The OL was defined as a line connecting the incisal tip of the most prominent maxillary central incisor (incision superius (is)) and the distobuccal cusp tip of the maxillary first molar. The MP was defined as a line connecting the Menton (Me) and Gonion (Go).

To standardize image sizes and account for magnification errors, digital pre-treatment (T0) and post-treatment (T1) cephalograms were uploaded to Adobe Photoshop CS (Adobe, San Jose, Calif) and resized to a 1:1 scale [21]. The inbuilt millimetric calibration ruler on the images was used as a guide to overlay the cephalograms and as a reference for linear magnification correction. These images were then printed on to professional printing paper using a 1200 × 2400 dpi color laser printer (Hewlitt-Packard, Palo Alto, Calif). Clear acetate paper (Great Lakes, Tonawanda, New York) was placed over the printed images, and manual tracing was carried out using a 0.3 mm lead pencil [22]. Linear measurements were carried out using digital calipers accurate to 0.01 mm (Neiko, Veneto, Italy), and a cephalometric protractor was used for angular measurements (OrthoPli Corporation, Philadelphia, Pennsylvania).

**Fig. 4:**
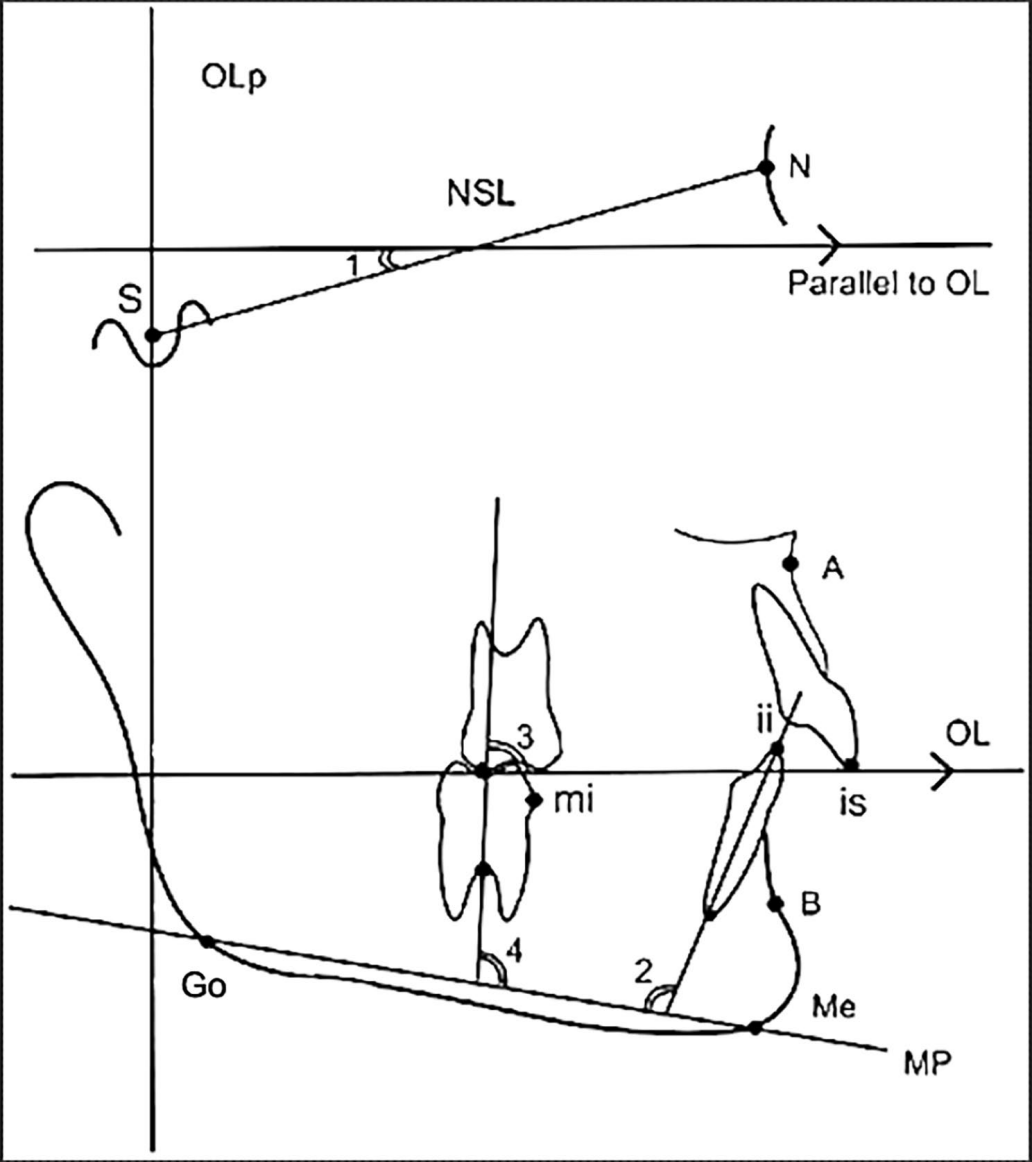
A representation of the lateral cephalogram tracing used for angular measurements. 1- NSL-OL°: angle between a parallel projection of the OL and the NSL line, 2- ii-MP°: angle between long axis of the most prominent mandibular central incisor and the mandibular plane, 3- Mi-OL°: angle between the long axis of the mandibular first molar and the occlusal line, 4- Mi-MP°: angle between the long axis of the mandibular first molar and the mandibular plane

In this study we used a modified sagittal-occlusal Pancherz analysis to create a grid for horizontal measurements [23]. A vertical reference plane was composed of a perpendicular from OL through Sella (Occlusal Line perpendicular (OLp)) at the T0 time point. The T0 and T1 tracings were then superimposed on the NSL, registered at the Sella, as per Steiner [24]. After superimposition, this vertical reference plane (OLp) was transferred from the T0 to the T1 tracing and used for the calculation of four horizontal linear measurements (Fig. 3). In addition, four angular measurements were also carried out at each time point, as seen in Fig. 4.

The following measurements assessed horizontal changes in the apical base: A-OLp, indicating the maxillary base position; and B-OLp, indicating the mandibular base position. The horizontal dental changes were evaluated by measuring the distance from the incisal tip of the most prominent mandibular incisor (incision inferius (ii)) and the most mesial point on the crown surface of the mandibular first molar (Mi) to the OLp, indicating mandibular central incisor and first molar position respectively. Rotation of the maxillary occlusal plane was denoted by the angular measurement between the OL and NSL.

Changes in angulation of the mandibular molar and incisor were characterized by angular measurements between the long axis of the most prominent mandibular incisor (ii) and MP, the long axis of the mandibular first molar (Mi) and MP, as well as between the long axis of the mandibular first molar (Mi) and the OL at each time point. The long axis of the mandibular incisor was defined as a line connecting the incisor tip and apex. The long axis of the mandibular first molar was defined as a line connecting the central fossa and furcation [25].

Additionally, we assessed the extent of changes in the canine relationship in each patient by comparing the digital scans or intraoral photographs of the side with the missing premolar at the T0 and T1 timepoints as previously performed by Metzner et al. [13]. A value of 0 mm was assigned to an Angle class I canine relationship, where the cusp tip of the maxillary canine lies in a cusp-embrasure relationship with the mandibular canine and first premolar buccally. The cusp tip of the maxillary canine served as a reference for loss of anchorage due to space closure by retraction of the mandibular anterior segment. Any corresponding deviation of the canine relationship to class II or III was assigned a corresponding negative or positive value. At the T1 time point, deviation of the mandibular canine from baseline in a distal direction was defined as loss of anchorage, and was assigned a negative millimetric value [13].

Finally, the efficiency of these appliances was evaluated by comparing the breakage and emergency visits for each type of appliance, as recorded on the clinical notes.

### Statistical analysis

Continuous variables were described using a mean and standard deviation, while categorical variables were summarized by count and percentage. The Q-Q plot/Shapiro-Wilk test (SAS PROC Univariate) was used to test for normality. The results indicated the normality assumption was not violated for the differences in each variable. The mean differences in the pre- and post-treatment measurements within the Herbst group, and within the TAD group, were analyzed using a paired t-test. A student t-test was used to compare differences in outcomes between the Herbst and TAD groups. *p* value < 0.05 was considered significant. Statistical analysis was performed with SAS 9.4 software (SAS Institute, Cary, NC, USA).

## Results

### Patient demographics

The Herbst group was made up of 21 patients (13 females and 8 males) with a mean age of 13.23 years (SD: 1.68 years). These patients presented with a total of 35 congenitally missing mandibular second premolars (14 bilateral, 7 unilateral). The TAD group, on the other hand, included 12 patients (9 females and 3 males) with an average age of 13.08 years (SD: 1.44 years). In this group, 15 sites (9 unilateral, 3 bilateral) of congenitally missing mandibular second premolars were recorded.

### Treatment related features

The total treatment time for the Herbst patients averaged 3.02 years (SD: 1.05 years). The Herbst device in these patients was in place for a mean of 11.8 months (SD: 2.99 months). All pre- and post-treatment measurements obtained from the group treated with the Herbst device are presented in Table 1. Within this group, the variables that showed a statistically significant change were the Mi-OLp (+ 7.69 mm, *p* < 0.01), B-OLp change (+ 2.26 mm, *p* < 0.01), ii-MP angulation (-3.59°, *p* = 0.01), Mi-OL angulation (-3.61°, *p* = 0.01), and Mi-MP angulation (-2.54°, *p* = 0.04). The other variables did not show a statistically significant difference between the pre- and post-measurements.

**Table 1:** Comparison of the Herbst and TAD measurements

Variables		Herbst Group				TAD Group				Comparison	
		Pre-Treatment	Post-Treatment	Difference	Significance (*P*-value)	Pre-Treatment	Post-Treatment	Difference	Significance (*P*-value)	Significance (*P*-value)	Confidence Interval (95%)
**A-OLp** (mm)	mean	72.13	72.06	-0.07	0.88	70.29	70.68	0.39	0.48	0.56	-2.09 1.15
	SD	4.75	4.79	2.28		7.24	6.40	1.90			
**ii-Olp** (mm)	mean	73.93	74.70	0.77	0.32	73.30	74.91	1.61	0.44	0.7	-5.46 3.78
	SD	5.17	5.95	3.47		10.24	6.18	6.99			
**Mi-Olp** (mm)	mean	51.39	59.09	7.69	< 0.01*	50.68	58.03	7.34	< 0.01*	0.75	-2.10 2.80
	SD	4.68	4.97	3.66		6.27	6.61	2.55			
**B-Olp** (mm)	mean	73.69	75.96	2.26	< 0.01*	73.28	74.27	0.99	0.28	0.26	-1.01 3.57
	SD	4.80	5.69	3.13		5.77	5.75	3.04			
**NSL-OL** (^0^)	mean	16.69	16.42	-0.26	0.77	18.20	19.62	1.41	0.42	0.34	-5.21 1.85
	SD	4.20	5.17	3.99		4.32	4.89	5.94			
**ii-MP** (^0^)	mean	92.92	89.33	-3.59	0.01*	92.16	89.62	-2.54	0.12	0.61	-5.24 3.13
	SD	6.10	6.69	5.86		7.98	8.79	5.29			
**Mi-OL** (^0^)	mean	85.59	81.97	-3.61	0.01*	83.20	84.25	1.04	0.61	0.07	-9.42 0.48
	SD	8.59	6.82	6.55		6.40	5.37	6.94			
**Mi-MP** (^0^)	mean	95.64	93.09	-2.54	0.04*	98.54	99.50	0.95	0.75	0.30	-10.28 3.65
	SD	6.27	6.59	5.42		7.95	6.80	10.49			
**E-Space** (mm)	mean	9.07				8.6				0.59	-1.86 0.92
	SD	1				2.86					
**Protraction rate** (mm/month)	mean	0.77				0.55				0.12	-0.57 0.11
	SD	0.51				0.26					
**Tx Time** (yrs)	mean	3.02				2.67				0.38	-1.10 0.43
	SD	1.05				1					
**Emergency visits rate** (visits/patient)	mean	1.47				0.25				< 0.01*	-1.85 -0.61
	SD	1.17				0.43					

*Statistically significant with *p* < 0.05

On the other hand, the mean total treatment time for the TAD group was 2.67 years (SD: 1 year), and TADs were in place for about 14.83 months (SD: 5.89 months). The pre- and post-treatment measurements of the TAD treated patients are also displayed in Table 1. We found that Mi-OLp was the only variable that showed a statistically significant change within this group (+ 7.34 mm, *p* < 0.01).

When comparing the findings of the Herbst to the TAD groups, we found that total treatment times were comparable between the two groups (*p* = 0.38). While there was a 4.65° difference in the post- to pre-treatment Mi-OL angle between the Herbst and TAD groups (-3.61° vs. 1.04°, respectively), this did not come to statistical significance (*p* = 0.07). Furthermore, no statistically significant differences were observed between the two groups for any of the other variables measured (Table 1).

We also found that while there were some significant differences between males and females in the pre-treatment measurements (A-OLp and B-Olp) and post-treatment measurements (A-OLp, ii-Olp, Mi-Olp, B-Olp and ii-Mp), the pre- to post-treatment comparison of males and females was not statistically significant (Table 2).

**Table 2:** Evaluation of differences in males and females measurements during Molar Protraction

Variables		Pre-Treatment		Difference	Significance (*P*-value)	Post-Treatment		Difference	Significance (*P*-value)	Comparison	
		Males	Female			Males	Females			Significance (*P*-value)	Confidence Interval (95%)
**A-OLp** (mm)	mean	74.77	69.82	-4.95	0.02*	74.66	70.02	-4.64	0.02*	0.71	-1.35
	SD	6.00	5.07	5.39		6.49	4.20	5.05			1.97
**ii-Olp** (mm)	mean	74.74	73.19	-1.55	0.64	77.87	73.24	-4.63	0.03*	0.22	-8.21
	SD	10.04	5.63	7.35		6.89	4.86	5.60			2.06
**Mi-Olp** (mm)	mean	53.28	50.07	-3.20	0.10	61.38	57.38	-4.00	0.04*	0.52	-3.28
	SD	4.10	5.49	5.08		5.94	4.96	5.29			1.68
**B-Olp** (mm)	mean	76.33	72.15	-4.18	0.02*	78.48	73.79	-4.69	0.02*	0.66	-2.89
	SD	4.65	4.81	4.76		6.81	4.42	5.31			1.87
**NSL-OL** (^0^)	mean	15.64	18.05	2.41	0.13	17.41	17.68	0.27	0.89	0.23	-5.71
	SD	3.85	4.29	4.15		4.91	5.50	5.32			1.44
**ii-MP** (^0^)	mean	94.95	91.50	-3.45	0.17	94.00	87.16	-6.84	0.01*	0.10	-7.49
	SD	5.98	6.92	1.54		7.66	6.24	6.73			0.72
**Mi-OL** (^0^)	mean	86.77	83.70	-3.07	0.30	83.05	82.86	-0.18	0.94	0.27	-2.33
	SD	6.15	8.52	7.83		6.44	6.46	6.45			8.10
**Mi-MP** (^0^)	mean	95.55	97.27	1.73	0.51	92.45	97.09	4.63	0.09	0.31	-2.87
	SD	5.72	7.56	7.02		8.90	6.18	7.17			8.69
**E-Space** (mm)	mean					9.18	8.76	-0.42		0.55	-1.84
	SD					2.19	1.72				1.00
**Protraction rate** (mm/month)	mean					0.76	0.66	-0.10		0.57	-0.45
	SD					0.39	0.49				0.25
**Tx Time** (yrs)	mean					3.37	2.67	-0.70		0.06	-1.45
	SD					1.21	0.87				0.04
**Emergency visits rate** (visits/patient)	mean					0.91	1.09	0.18		0.68	-0.72
	SD					0.94	1.31				1.09

*Statistically significant with *p* < 0.05

### Canine relationship outcomes

**Fig. 5:**
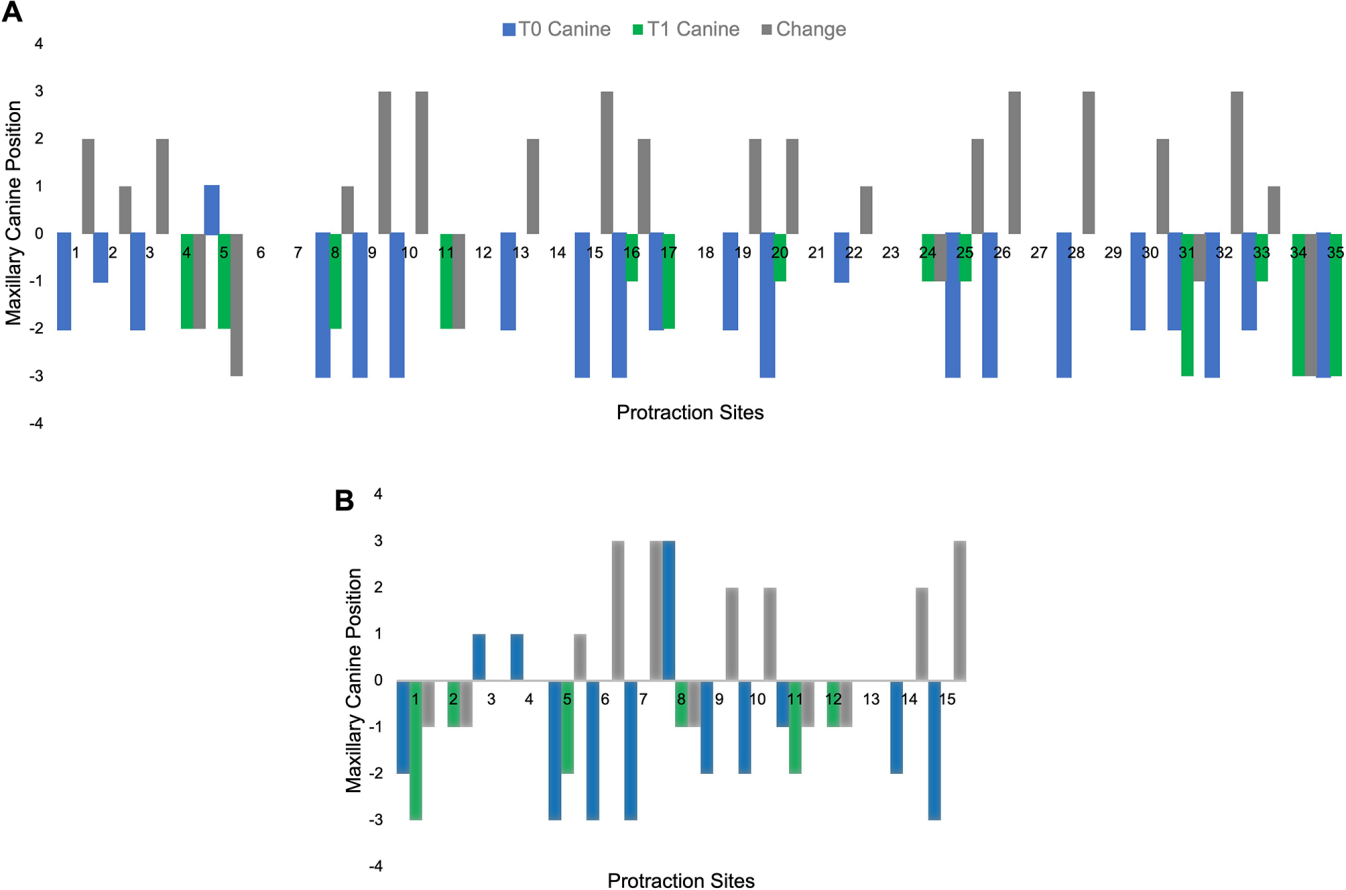
Canine relationship changes in the Herbst (**A**) and TAD (**B**) groups. The Y-axis denotes the millimetric position of the maxillary canine, where class I is 0 mm, and a shift towards a class II or class III is denoted by a negative or positive value respectively. The X-axis groups the initial, final and net change in the canine relationship per protraction site

In the Herbst group, for the 35 sites with missing mandibular second premolars, the mean distal occlusion at T0 was − 1.43 mm (SD = 1.36 mm). In 82.85% of these sites, the canine relationship was maintained or improved toward a class I occlusion by a mean of 1.31 mm (SD = 1.19 mm) (Fig. 5A). On the other hand, 17.14% of Herbst cases displayed canine relationships that worsened toward class II. We found that mean loss of anchorage was − 1.83 mm (SD = 0.75 mm). We also examined the 15 missing premolar sites in the TAD group, which had a mean distal occlusion that was − 1.07 mm (SD = 1.83 mm) at T0. The canine relationship in this group was maintained or improved toward a class I in 66.7% of these sites, by a mean of 1.6 mm (SD = 1.26 mm) Within the TAD sample, 33.33% showed a worsening of canine relationships towards a class II, with a mean change of -1 mm (SD = 0) (Fig. 5B).

### Summary of emergency visits

Out of the 21 patients in the Herbst group, 80.1% presented with emergencies, accounting for a total of 31 visits (mean = 1.47 emergency visits per patient). Bond failure leading to loosening of appliances and appliance breakages accounted for 50% of these emergency visits. Other reasons for these visits included lingual or buccal irritation, loose Herbst arms and loose power chains. In the TAD group approximately 25% of the 12 patients presented with a total of 3 emergency visits (mean = 0.25 emergency visits per patient). One TAD failure was reported 3 months after placement and was replaced. Protraction was completed and the TAD was removed 11 months after initial placement. The other two emergency visits related to loose ties to the TAD. This difference in the occurrence of emergency visits was statistically significant (*p* < 0.01) (Table 1).

## Discussion

The utilization of the Herbst device as a fixed functional appliance is well documented, with reported treatment effects such as slight inhibition of the forward movement of the maxilla, protrusion and proclination of the mandibular incisors, and a forward movement of the mandible [26, 27]. Within our Herbst group, -0.07 mm of change was observed in the maxillary base position (A-OLp). The literature indicates there tends to be a small decrease in the A-OLp value with the Herbst, indicative of a restriction in anterior movement of the maxilla [28]. This has been reported to vary between − 0.26 to -0.70 mm [28]. In comparison, our TAD group demonstrated a + 0.39 mm change in the A-OLp which is consistent with control groups in Herbst studies which showed 0.7 to 1.4 mm of anterior maxillary movement [28]. In this study we noted a 2.26 mm forward movement of the mandible in the Herbst group. This agrees with previously published literature on the Herbst which have reported a + 0.7 mm to 3.7 mm change in mandibular length. The TAD group showed 0.99 mm of mandibular change within the treatment period, conforming to the 0.5 –2.2 mm range in control groups in Herbst studies [28]. A meta-analysis by Yang et al., studying the effectiveness of the Herbst device proposed the sagittal change in the mandibular position was a result of change in condylar position as well as a net increase in mandibular length [28]. The contribution of mandibular growth toward these findings must be kept in mind, especially since the included patients approximate the age range for the pubertal growth spurt [29]. Overall, a combination of these maxillary and mandibular effects of the protraction Herbst imply an additional benefit to this device- aiding in skeletal class II correction compared to TADs.

Due to the anterior force vector applied on the mandibular dentition, one of the effects of the Herbst device is proclination of the mandibular incisors. Proclination between 2.5 and 10.4 degrees have been reported immediately after Herbst removal, however a relapse of 8 degrees was demonstrated by VanLaecken et al. [27] 16 months post Herbst removal, bringing the overall proclination to 2.4 degrees. A net retroclination of the mandibular incisor position was noted at the end of treatment in our Herbst group, but did not reach statistical significance. Since we did not have access to cephalograms taken immediately after Herbst removal, we theorize this change could correspond to a relapse after the removal of the Herbst device [27, 30], and also a loss of anterior anchorage due to space closing mechanisms utilized while protracting the mandibular molar [31, 32]. Similarly, a slight uprighting of the mandibular incisors was observed in the TAD group. Al-nimri et al., discussed mandibular incisor position following premolar extractions, and pointed out that the initial crowding and incisor proclination as well as the amount of space closure required should be taken into account when evaluation final incisor proclination [31]. We did not consider the amount of crowding in our samples. Thus, future studies taking this variable into account could give a clearer picture of causality of the changes.

In our Herbst group, canine relationships were maintained or improved in 82.5% of patients by 1.31 mm. This improvement aligns with the primary function of the Herbst, which is to advance the mandible sagittally. About a third of the TAD sample demonstrated a worsening of canine relationships, compared to less than one fifth of the Herbst cases. A similar study by Metzner et al. [13] compared the effectiveness of molar protraction using the Herbst and Temporary anchorage devices. The results found a 90.9% improvement in occlusion toward a class 1 canine relationship in their Herbst sample, while this improvement was only noted in 14.3% of their TAD group. Incisor proclination, which is a side-effect of the Herbst device, contributes to an additional anterior anchorage to counteract the lingual tipping side effect of space-closing mechanics [13]. A combination of skeletal and dental change contributes to a tendency to improve the dental relationship, especially in circumpubertal populations [28, 29].

Variation in the bone densities between the maxilla and mandible results in a slower rate of tooth movement in the mandible [33]. Presence of a thicker, 2 mm cortical plate in the mandible, compared to 1.5 mm in the maxilla, and dense radial bony trabeculae contribute toward an increased resistance to tooth movement. However, the analysis of the differences in bone thickness was not an aim of this study and we only evaluated tooth movement in the mandible after primary molar extractions. We showed that the rates of molar protraction displayed no statistically significant differences between groups and were recorded as 0.55 mm/month for the TAD patients, and 0.77 mm/month for the Herbst ones, which also correspond to literature reports [34, 35]. A randomized clinical trial by Dixon et al., demonstrated that the rate of orthodontic space closure varied between 0.35 mm/month to 0.85 mm/month depending on the type of space closing mechanics used (power-chain, nickel titanium springs or active tie backs) [34, 35]. The rate of mesial translation of molars using TADs has been reported to vary between 0.32 mm/month to 0.35 mm/month [8, 34, 35]. On the other hand, Metzner et al. observed that Herbst supported molar protraction averaged 0.51 mm/month [13].

Applying the protraction force to the crown of the mandibular molar (occlusal to its center of resistance) creates a tendency to tip the tooth mesially and rotate it. A finite element analysis of mandibular molar protraction demonstrated that application of a lingual force from the molar crown to the premolar crown decreases this rotational tendency, but causes an increase in the mesio-distal tipping [36]. The observed mesial molar tipping in the Herbst group surpassed our initial expectations, demonstrating a statistically significant a net change of -2.54° and − 3.61° in the angulation between mandibular first molar and the mandibular and occlusal plane, respectively. Addition of a gable bend on the lingual framework to create a counterclockwise moment, or using a thicker wire to reduce slop between the lingual arch and the molar slot could help mitigate this tipping [14].

In a growing patient, the growth of the dentoalveolar segments is the primary determinant of the occlusal plane inclination, while the position of the occlusal plane is based mainly on the vertical position of the maxillary teeth [37, 38]. During orthodontic intervention, this occlusal plane can be altered by molar mesialization, and vertical position changes of the molars and incisors [37, 38]. Li et al. reported a link between occlusal plane changes after orthodontic intervention and dentoskeletal patterns [39]. They noted an increase in the bisected occlusal plane to SN angle after treatment in patients with a class II skeletal pattern, while no significant changes were seen in the class I and III groups. Clockwise rotation of the maxillary occlusal plane in relation to the Sella-Nasion line with the use of the Herbst has been documented, but a relapse of this rotation is noted after device removal [27, 30]. The use of Class II intermaxillary elastics for correction of an anterior-posterior discrepancy, or for anchorage reinforcement, can also result in a clockwise rotation of the occlusal plane due to mandibular molar and maxillary incisor extrusion [37]. In our current study, no significant changes in the occlusal plane angulation were noted within either group, and no significant difference existed between the groups.

In comparing the occurrence of emergency visits, we noted a significant difference between the Herbst and TAD groups (*p* < 0.01). Emergency visits were observed 3.2 times more often in the Herbst patient sample than in the TAD sample. A total of 31 emergency visits were noted in the Herbst-treated group. About 50% of these were related to bond failures and appliance breakages. These findings are in agreement with published data, wherein complication rates of up to 88% have been reported in Herbst patients [40, 41]. The emergency types have been noted to be independent of whether the Herbst is designed as a removable mandibular acrylic splint or a lower cantilever [40, 41]. Loosening of the Herbst screws and distortion of the rods were reportedly the most common cause of emergencies in the lower cantilever type of Herbst [42], which parallel the findings of this study. In contrast, 1 of the 3 emergency visits for our TAD group related to TAD failure, while the other 2 were caused by loose ligature ties to the TAD. Success of miniscrews placed between the mandibular canine and first premolar premolar has been reported as 12.3% [42]. A lower failure rate is associated with indirect anchorage (8.6%) as compared to direct anchorage (9.9%). Longer miniscrews allow for more mechanical retention and presumably greater success. Age has also been reported to be a factor in the success of TADs, with patients aged > = 20 years showing better success rates potentially due to a difference in the bone quality and quantity [42]. However, the difference in protraction rates and treatment times between the Herbst and TAD groups in our study of adolescent patients did not reach statistical significance, suggesting that the emergencies, if addressed in a timely manner, do not compromise overall outcomes.

Assessing the utilization of the Herbst device in molar protraction, our findings indicate that the Herbst device can be a useful clinical tool to aid in anchorage control while protracting mandibular molars. With the advantage of improvement in sagittal skeletal relationships, it can be considered for molar protraction in growing patients with a mandibular deficiency. Comparing the effectiveness of protraction using the Herbst appliance and TADs, molar protraction, regardless of the adjunct appliance used, is a long process. Total treatment time was approximately 3 years for both groups. This should be considered while making decisions regarding protraction versus prosthetic replacement. No statistical differences were noted in comparing the rate of molar protraction between the groups. Mesial molar tipping was noted in both groups, and needs to be managed clinically. However, the Herbst group had to deal with more emergency visits than the TAD group. Overall, this data can aid orthodontic practitioners in determining the best treatment modality for their specific patient populations. The Herbst device could be a viable alternative in cases of retrognathia with upright maxillary incisors and missing mandibular second premolars, or if parents are resistant to their child receiving temporary anchorage devices.

The findings of this study should be considered in light of certain limitations, some of which have already been discussed. Another one is that this was a retrospective study and dependent on entries into clinical databases in three different offices. Differences in treatment approaches of the doctors could have impacted the results. The stringent exclusion criteria resulted in a relatively small sample size that could have influenced estimates, and potentially resulted in an increase in type II errors. Age, type of initial malocclusion and degree of crowding were also not considered. Consequently, the results may not be generalizable to the populations. Our study can be viewed as a preliminary guide to higher powered research in the future. Furthermore, this study was based on cephalometric images, which are two-dimensional presentations of three-dimensional subjects. The radiographs used were taken on different machines and adjusted for magnification errors. However, a study based on CBCT images for three-dimensional analysis would improve accuracy. Moreover, changes in the vertical and transverse dimensions were not part of our measurements, but should be part of future studies. Immediate post-protraction cephalometric data was not available, and final molar tip could have been affected by bracket repositioning after mesialization was complete. Similarly, the Herbst and TADs could have been left in place even after protraction was complete to stabilize antero-posterior change of the molar, or as a failsafe to address potential relapse of the E-space. The use of Class II elastics during or after protraction was not recorded, and could have influenced final incisor position. Another limitation is the lack of model analysis to complement cephalometric data. Finally, our subjects were all growing individuals, but the effect of mandibular growth was not considered in this study. Variations in growth patterns could impact the results. Additional superimpositions on the mandible would help give us a more complete understanding of what changes could be attributed to growth and help quantify these changes.

## Conclusions

The effectiveness of protraction using the Herbst device and TADs are comparable. Mesial tipping of the mandibular molar is a side effect of space closing mechanics that needs to be managed. Both treatment modalities present excellent means to address missing mandibular second bicuspids. The Herbst could potentially present an advantage in terms of anterior anchorage control due to mandibular sagittal advancement and incisor proclination. However, more emergency visits were noted in patients treated with the Herbst appliance than those treated with TADs.

## Data Availability

The datasets used and/or analyzed during the current study are available from the corresponding author on reasonable request.

## Abbreviations

TAD: Temporary Anchorage Device
TA: Tooth Agenesis
NSL: Nasion-Sella Line
OL: Occlusal Line
MP: Mandibular Plane
Me: Menton
Go: Gonion
OLp: Occlusal Line Perpendicular
Ii: incision inferius
Is: Incision superius
Mi: Mandibular first molar
